# Determination of Polyphenol Components of Korean Prostrate Spurge (*Euphorbia supina*) by Using Liquid Chromatography—Tandem Mass Spectrometry: Overall Contribution to Antioxidant Activity

**DOI:** 10.1155/2014/418690

**Published:** 2014-03-03

**Authors:** Yi Song, Sung Woo Jeong, Won Sup Lee, Semin Park, Yun-Hi Kim, Gon-Sup Kim, Soo Jung Lee, Jong Sung Jin, Chi-Yeon Kim, Ji Eun Lee, Se Yun Ok, Ki-Min Bark, Sung Chul Shin

**Affiliations:** ^1^Department of Chemistry and Research Institute of Life Science, Gyeongsang National University, Jinju 660-701, Republic of Korea; ^2^Department of Internal Medicine, Institute of Health Sciences and Gyeongnam Regional Cancer Center, Gyeongsang National University, Jinju 660-702, Republic of Korea; ^3^Research Institute of Life Science and College of Veterinary Medicine, Gyeongsang National University, Jinju 660-701, Republic of Korea; ^4^Department of Food and Nutrition, Institute of Agriculture and Life Science, Gyeongsang National University, Jinju 660-701, Republic of Korea; ^5^Division of High Technology Materials Research, Busan Center, Korea Basic Science Institute (KBSI), Busan 618-230, Republic of Korea; ^6^Department of Dermatology Institute of Health Science, Gyeongsang National University Hospital, Jinju 660-702, Republic of Korea; ^7^Department of Chemical Education and Research Institute of Life Science, Gyeongsang National University, Jinju 660-701, Republic of Korea

## Abstract

The Korean prostrate spurge *Euphorbia supina* is a weed that has been used in folk medicine in Korea against a variety of diseases. Nine polyphenols were characterized for this plant by using high-performance liquid chromatography-tandem mass spectrometry (HPLC-MS/MS) and the results were compared with the literature data. The individual components were validated using the calibration curves of structurally related external standards and quantified for the first time by using the validated method. Correlation coefficients (*r* ^2^) were >0.9907. The limit of detection and limit of quantification of the method were >0.028 mg/L and 0.094 mg/L, respectively. Recoveries measured at 50 mg/L and 100 mg/L were 76.1–102.8% and 85.2–98.6%, respectively. The total amount of the identified polyphenols was 3352.9 ± 2.8 mg/kg fresh plant. Quercetin and kaempferol derivatives formed 84.8% of the total polyphenols. The antioxidant activities of the flavonoids were evaluated in terms of 1,1-diphenyl-2-picrylhydrazyl and 2,2′-azinobis(3-ethylbenzothiazoline-6-sulfonic acid) radical cation-scavenging activity, and the reducing power showed a dose-dependent increase. Cell viability was effectively suppressed at polyphenol mixture concentrations >250 mg/L.

## 1. Introduction

The Korean prostrate spurge* Euphorbia supina* is a weed that belongs to the Euphorbiaceae family and is native to North America. It is found in poor, drought-stressed turf and grows well during hot, dry weather in thin soils. It sprouts purple-spotted, up to 0.60-in oval leaves, and blooms very small, inconspicuous flowers in the summer [1].

The plant has been used in folk medicine in Korea against a variety of conditions such as diarrhea and suppurated swelling and as a styptic [2]. It was reported that the plant contains a number of biologically interesting organic substances, including terpenoids [3, 4], tannins, and polyphenols [5–7].

Of all bioactive natural constituents, polyphenols have attracted a great deal of interest, because they have beneficial effects to human health. Epidemiological studies have shown that polyphenols render many biological benefits, including a reduced risk of chronic diseases [8, 9] and antioxidant, antiaging, and antimicrobial properties [10]. In the plants, polyphenols function as physiologically active substances, such as attractants, feeding deterrents, materials used to communicate with the surrounding environment, and materials used as defense against biotic and abiotic stresses [11, 12]. Although the pharmaceutical efficacy of *E. supina* could be ascribed, at least partly, to the polyphenols, few studies have been conducted to validate this [3, 13].

The objective of the present study was to comprehensively characterize the polyphenol metabolomes of Korean *E. supina* by using high-performance liquid chromatography-tandem mass spectrometry (HPLC–MS/MS) and to investigate their biological benefits, including antioxidant and hepatoprotective effects. HPLC–MS/MS is a useful technique for analyzing plant polyphenols, because it provides online structural information and characterizes unknown substances even when no reference standards are available [14].

## 2. Materials and Methods

### 2.1. Materials and Chemicals

*E. supina* was purchased in mid-April 2012 from a market in Jinju, South Korea. The plant was authenticated by Professor Moo Ryong Huh, a plant taxonomist with the Research Institute of Agricultural Life Science, Gyeongsang National University. A voucher plant was deposited in the herbarium at this institute. The plant was washed with water, lyophilized, and stored in dark containers at −70°C until needed. All chemicals were purchased from Sigma-Aldrich Co., LLC (St. Louis, MO, USA). Gallic acid, protocatechuic acid, 7-hydroxycoumarin, quercetin 3-*O*-glucoside, and kaempferol, which were purchased from Sigma-Aldrich Co., LLC (St. Louis, MO, USA), were used as external standards after recrystallization in ethanol. The purity of all standards was confirmed to be >99% by using HPLC. All solvents and water were obtained from Duksan Pure Chemicals Co., Ltd. (Ansan, Republic of Korea).

### 2.2. Extraction and Purification

The lyophilized *E. supina* tissue (10 g) was ground into powder and extracted in ethyl acetate (300 mL) at 80°C for 20 h. The extract was filtered through a Büchner funnel and concentrated at reduced pressure under 40°C by using a rotator evaporator. The concentrated solution was washed with *n*-hexane (100 mL × 3), extracted with ethyl acetate (100 mL × 3), and dried over anhydrous sodium sulfate (Na_2_SO_4_). The solvent was removed under reduced pressure. The sticky residue was placed on top of a silica gel sorbent (3 × 1.7 cm i.d.) and eluted using a mixture of methanol:dichloromethane (1 : 5, 25 mL). The solvent was removed to give a mixture of polyphenols (0.9% of the dried plant). The mixtures were reconstituted in ethyl acetate (0.03 g/mL), filtered through 0.45 *μ*m cellulose membranes, transferred into silanized vials, and stored at −20°C until analysis.

### 2.3. HPLC–MS/MS

HPLC–MS/MS experiments were conducted according to a previously reported method [15], except for the use of a solvent system consisting of 0.5% aqueous formic acid (A) and methanol (B). The gradient conditions of the mobile phase were from 10 to 30% B over 10 min, increased to 90% B over 40 min, and increased again to 98% B over 5 min.

### 2.4. Quantification and Validation

All components were quantified using chromatograms obtained at 254 nm. The quantification was validated in terms of linearity, limit of detection (LOD), limit of quantification (LOQ), accuracy, and precision.

The individual components for which standards were not available, except for gallic acid (**1**) and protocatechuic acid (**2**), were quantified using the calibration curves of structurally related external standards. Thus, nodakenin (**3**) was quantified as 7-hydroxycoumarin, quercetin derivatives (**4**, **5**, **8**) as quercetin 3-*O*-glucoside, and kaempferol derivatives (**6**, **7**, **9**) as kaempferol. Plant polyphenols can be quantified using a standard curve of structurally related compounds [16]. A stock solution of each standard (10 mg/L) was prepared by dissolving the appropriate amounts in methanol and storing at −20°C. Linearity was assessed using six different concentrations, (1, 10, 50, 100, 1000, and 2000 mg/L) of each standard and by plotting the concentration of the standard against the peak area. LOD and LOQ were determined by injecting each standard solution into the HPLC until the signal-to-noise ratio for the standards reached 3 : 1 and 10 : 1, respectively. The accuracy of the methods was estimated as recovery = A/IS-C/B/IS-C, where A is the peak area obtained for the analyte spiked preextraction, B is the area obtained for the analyte spiked after extraction, and C is the area of the blank extraction. The precision of the method was represented as a relative standard deviation (RSD).

### 2.5. Antioxidant Activity Measurement

A series of methanol solutions of the *E. supina* polyphenol mixture (25, 50, 100, 200, and 500 mg/L) were prepared and used for the antioxidant assay. Antioxidant activities were measured in terms of 1,1-diphenyl-2-picrylhydrazyl radical (DPPH^*∙*^) and 2,2′-azinobis(3-ethylbenzothiazoline-6-sulfonic acid) (ABTS^*∙*+^) radical cation-scavenging activity and reducing power (RP) assay according to a method reported in our previous studies [16].

### 2.6. Effects of the Polyphenol Mixture of *E. supina* on Hep3B Cell Viability

#### 2.6.1. Cell Viability Assay

Hepatic cancer cells (1 × 10^4^ cells per well) were plated onto 12-well plates and treated with the polyphenol mixture of *E. supina* at concentrations of 31.25, 62.5, 125, 250, and 500 mg/L or vehicle (dimethyl sulfoxide, DMSO) alone for 24 h. Cell viability was estimated by measuring the 3-(4,5-dimethylthiazol-2-yl)-2,5-diphenyltetrazolium bromide (MTT) metabolism. Thus, 100 *μ*L of MTT solution (5 mg/L) was added to each well of a 12-well plate, and the cells were maintained for 3 h at 37°C. After the supernatant was removed, the remaining violet residue was dissolved in DMSO (1 mL). The absorbance values were measured using a microplate reader at 540 nm. Cell viability was expressed as a percentage of proliferation versus controls, which was set at 100%.

#### 2.6.2. Cell Morphology Observation

Hep3B cells (5 × 10^4^ cells per well) were plated onto 6-well plates and treated with the polyphenol mixture of *E. supina* at concentrations of 31.25, 62.5, 125, 250, and 500 mg/L or vehicle alone for 24 h. Cell morphological change was observed under an optical microscope (Olympus CKX 41, Japan).

### 2.7. Statistical Analysis

All statistical analyses were performed according to a method described previously [16].

## 3. Results and Discussion

### 3.1. Separation and Characterization

**Figure 1:**
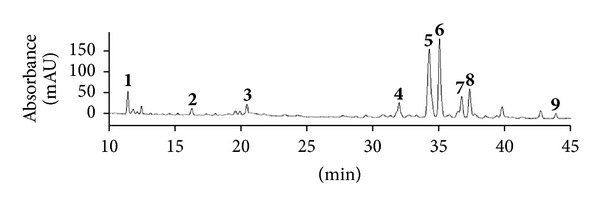
Chromatograms of the* Euphorbia supina *polyphenol mixture obtained using high-performance liquid chromatography:**1**, gallic acid; **2**, protocatechuic acid; **3**, nodakenin; **4**, quercetin 3-*O*-hexoside; **5**, quercetin 3-*O*-pentoside; **6**, kaempferol 3-*O*-hexoside; **7**, kaempferol 3-*O*-pentoside; **8**, quercetin; **9**, kaempferol. Detection wavelength: 254 nm.

**Figure 2:**
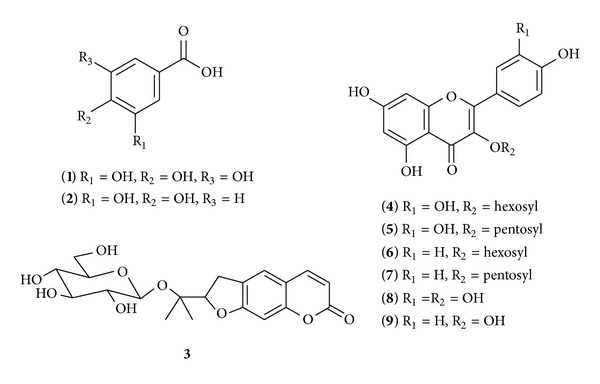
Structures of the eight polyphenols and one nodakenin in *Euphorbia supina. *

A mixture of polyphenols was isolated from *E. supina* by methanol extraction at 80°C, followed by elution in ethyl acetate over a silica gel cartridge. The polyphenols were characterized through HPLC by using a C_18_ column, MS/MS in negative- and positive-ion modes, and comparison with the previous literature data. The HPLC chromatograms of the plant are shown in Figure 1. Nine polyphenols were labeled in the 10 to 45 min retention time segments of the chromatograms. The structures and HPLC–MS/MS data of the nine polyphenols are shown in Figure 2 and Table 1, respectively.

Polyphenol **1** was identified as gallic acid. Its MS/MS spectrum produced a [M–H]^−^ of *m/z* 169, which fragmented to yield 125 [M–H–CO_2_]^−^ and 97 [M–H–CO_2_–CO]^−^ [17]. Polyphenol **2** was identified as protocatechuic acid. Its MS/MS consisted of [M–H]^−^ at *m/z* 153 and 109 [M–H–CO_2_]^−^ [17]. Component **3** was identified as nodakenin. Its MS/MS spectrum produced a [M+H]^+^ of *m/z* 409, which fragmented to 247 [M+H–glucosyl]^+^, 229 [M+H–glucosyl-H_2_O]^+^, and 203 [M+H–glucosyl–CO_2_]^+^ [18]. Polyphenol **4** yielded [M–H]^−^ of *m/z* 463, which fragmented to 301 [M–H–hexosyl]^−^, 283 [M–H–hexosyl–H_2_O]^−^, and 255 [M–H–hexosyl–H_2_O–CO]^−^. Polyphenol **4** was quercetin 3-*O*-hexoside [16, 19]. The MS/MS spectrum of polyphenol **5** consisted of [M–H]^−^ at *m/z* 433, 301 [M–2H–pentosyl]^−^, and 273 [M–2H–pentosyl–CO]^−^; polyphenol **5** was identified as quercetin 3-*O*-pentoside [20]. Polyphenol **6** gave [M–H]^−^ at *m/z* 447, which produced 285 ([M–H]^−^–hexosyl) and 255 ([M–H]^−^–hexosyl–H_2_). It was identified as kaempferol 3-*O-*hexoside [16, 21]. Polyphenol **7** was identified as kaempferol 3-*O*-pentoside. Its MS/MS consisted of [M+H]^+^ at *m/z* 419 and 287 [M+H–pentosyl]^+^ [22, 23]. Polyphenol **8** yielded [M+H]^+^ at *m/z* 302, which was fragmented to typical fragment ions 273 [M+H–CO]^+^, 179 [M–CC_6_H_5_O_2_]^+^, and 153 [C_7_H_5_O_4_, retro-Diels-Alder fragment]. Polyphenol **8** was quercetin [24, 25]. Polyphenol **9** was identified as kaempferol and gave [M+H]^+^ at *m/z* 287, which produced 257 [M+H–CO]^+^ and 153 [C_7_H_5_O_4_, retro-Diels-Alder fragment] [26, 27].

### 3.2. Quantification

The nine polyphenols identified in the Korean *E. supina *were quantified for the first time from peak areas of the LC-UV chromatogram obtained at 254 nm. Quantification was validated based on representative external standards from the same group. The validation data are listed in Table 2. Regression equations were prepared in the form of *y* = a*x* + b, where *y* and *x* were the peak area and the concentration of each standard, respectively. The regression analysis showed correlation coefficients (*r* ^2^) > 0.9907 for all five standards (*n* = 5), indicating good linearity. The LODs of the method were 0.028–0.142 mg/L and LOQs were 0.094–0.473 mg/L, indicating good performance limits. Recoveries measured at 50 mg/L and 100 mg/L were 76.1–102.8% and 85.2–98.6%, respectively. Precisions of the method at 50 mg/L and 100 mg/L were 0.1–6.1% and 0.5–14.0%, respectively. Both the accuracy and precision values were acceptable.

The contents of individual components are listed in Table 3. The total amount of the identified polyphenols was 3352.9 ± 2.8 mg/kg fresh plant. Quercetin and kaempferol derivatives formed 84.8% of the total polyphenols. The plant comprised quercetin 3-*O*-pentoside (**5**) as the most dominant component, followed by kaempferol 3-*O*-hexoside (**6**). Quercetin, kaempferol, and their sugar-bound derivatives are major representatives of the polyphenol subclass that display the antioxidant activity to scavenge reactive oxygen species. As a result, *E. supina*, which is rich in such components, could be effective for reducing the risk of various chronic diseases resulting from oxidative damage, such as cancer, atherosclerosis, and inflammation [27, 28].

### 3.3. Antioxidant Activity

The polyphenol mixture isolated from Korean *E. supina* was evaluated for its antioxidant effects. Contemporary interest in polyphenols focuses on the epidemiological association between their potent antioxidant properties and a low incidence of chronic diseases. Epidemiological studies have shown that oxidative stress plays an important role in the pathogenesis of various chronic diseases, including cancer, cardiovascular disease, atherosclerosis, hypertension, diabetes, neurodegenerative disorders, rheumatoid arthritis, and aging [29–31]. Polyphenols can reduce oxidative stress and thus might protect and/or retard disease development [32, 33]; therefore, it is necessary to evaluate the antioxidant properties of the polyphenols in medicinal herbs.

Antioxidant capacity can be evaluated by using a number of *in vitro* methods. Because the assay results are method dependent, a combined assay involving several methods is often used [34]. In this study, the antioxidant activity of the polyphenol mixture isolated from *E. supina* was determined by DPPH^*∙*^ and ABTS^*∙*+^ scavenging and RP assay at a concentration ranging from 25 to 500 mg/L. In DPPH^*∙*^ scavenging tests, antioxidant activity is monitored by measuring the disappearance of purple DPPH^*∙*^, which can be detected spectrophotometrically at 517 nm [16]. In the ABTS^*∙*+^ scavenging assay, the added antioxidants reduce the deep blue ABTS^*∙*+^ to ABTS, and the decrease in absorbance of ABTS^*∙*+^ at 414 nm is monitored [16]. The RP assay can also serve as an indicator of antioxidant activity. The added antioxidants convert the iron ion (Fe^3+^) to Fe^2+^. The increase in absorbance of the deep-green Fe^2+^ solution at ~700 nm is monitored [16]. The assay results are provided in Table 4. The antioxidant capacity assayed using three methods showed a similar tendency. Thus, the antioxidant capacity of the *E. supina* polyphenol mixture showed a dose-dependent increase. DPPH^*∙*^ or ABTS^*∙*+^ scavenging activity can be represented as an EC_50_ value, which is the antioxidant concentration required to bring about a 50% loss in absorbance at 517 nm for DPPH^*∙*^ and 414 nm for ABTS^*∙*+^ as determined by linear regression analysis [16]. RP can be represented as EC_0.3_, which is the reducing activity presented by the sample concentration at 0.3 of the absorbance value at 700 nm. Low EC_50_ and RP values signify high antioxidant activity [33]. The DPPH^*∙*^ and ABTS^*∙*+^ scavenging activity of butylated hydroxytoluene (BHT) as the control were 121.85 ± 0.39 mg/L and 93.85 ± 0.43 mg/L, respectively. The EC_0.3_ value of BHT was 26.71 ± 0.69 mg/L. The antioxidant capacity values represented in terms of the DPPH^*∙*^ and ABTS^*∙*+^ scavenging activity and RP value were lower than those of BHT (*P* < 0.05).

### 3.4. Growth Inhibitory and Morphological Effects of Polyphenol Mixture of Korean *E. supina*

**Figure 3:**
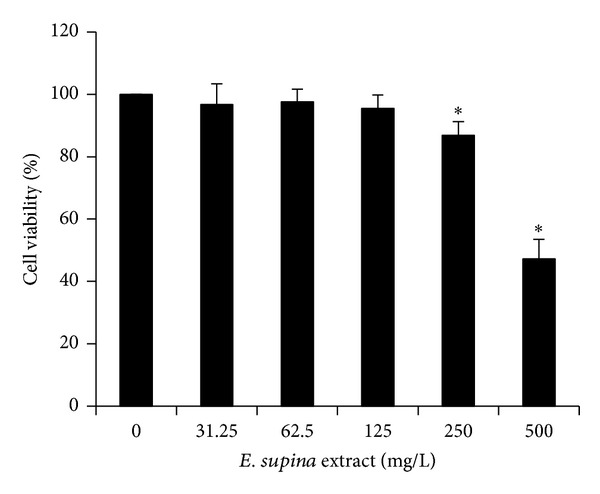
Antiproliferation effect on Hep3B cells by the *Euphorbia supina *polyphenol mixture. Hep3B cells were treated with the indicated concentrations of the *E. supina *polyphenol mixture for 24 h and viability was determined using 3-(4,5-dimethylthiazol-2-yl)-2,5-diphenyltetrazolium bromide (MTT) assay. Data represent the mean ± standard deviation (SD) of three replicates of independent experiments. The asterisk (∗) indicates a significant difference from the control group (*P* < 0.05).

**Figure 4:**
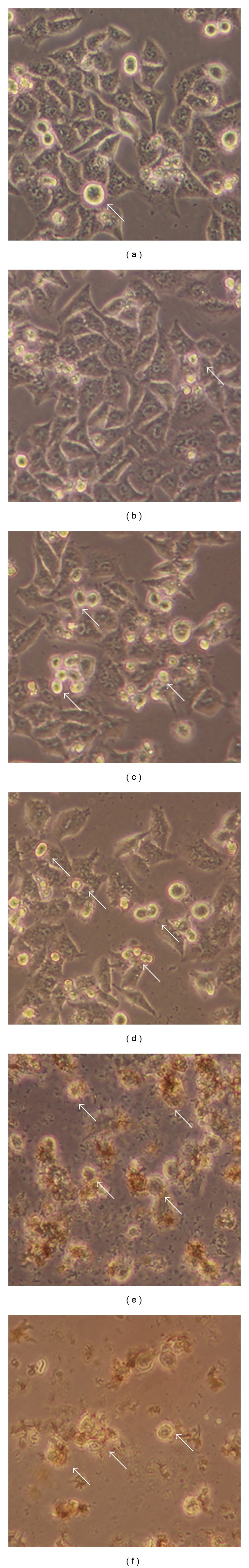
Morphological changes in Hep3B cells. Morphology of Hep3B cells visualized by optical microscopy (×100). The cells were treated with various concentrations of the *Euphorbia supina *polyphenol mixture for 24 h. (a) Control, (b) 31.25 mg/L, (c) 62.5 mg/L, (d) 125 mg/L, (e) 250 mg/L, and (f) 500 mg/L. White arrows indicate suspended cells.

The anticancer activity of the polyphenol mixture of Korean *E. supina* was evaluated for human hepatocellular carcinoma Hep3B cells by MTT assay. The assay is a colorimetric method that measures cancer cell viability, quantifying the activity of the mitochondrial enzyme that reduces the yellow MTT molecule to purple formazan [34]. The cell line was incubated with serial concentrations of the polyphenol mixture ranging from 31.25 to 500 mg/L for 24 h and then subjected to MTT assays. The results are shown in Figure 3. The cell viability was decreased at polyphenol mixtures >250 mg/L, and the IC_50_ value was 500 mg/L. After treatment with the polyphenol mixtures for 24 h, the morphological changes such as loss of cell adhesion and floating cell debris were also observed, as shown in Figure 4. The MTT assay results and morphological changes show that the polyphenol mixtures effectively suppressed cell viability.

## 4. Conclusion

Nine polyphenols from the Korean *E. supina* were profiled using a single HPLC–MS/MS run. The antioxidant activities of the flavonoids were evaluated in terms of DPPH^*∙*^ and ABTS^*∙*+^ scavenging activities, and the reducing power showed a dose-dependent increase. Suppression of cell viability was observed at polyphenol mixture concentrations >250 mg/L.

## Figures and Tables

**Table 1:** Mass spectral data of the *Euphorbia supina* polyphenol mixture.

Compounds	[M − H]^−^/[M + H]^+^	MS/MS	References
Gallic acid (**1**)	169	169, 125, 97	[17]
Protocatechuic acid (**2**)	153	153, 109, 108	[17]
Nodakenin (**3**)	/409	409, 391, 353, 389, 247, 229, 203, 185	[18]
Quercetin 3-*O*-hexoside (**4**)	463	463, 301, 300, 283, 271, 255, 151	[16, 19]
Quercetin 3-*O*-pentoside (**5**)	433	433, 300, 273, 271, 255, 179, 151	[20]
Kaempferol 3-*O*-hexoside (**6**)	447	447, 285, 255	[16, 21]
Kaempferol 3-*O*-pentoside (**7**)	/419	419, 309, 287, 155	[22, 23]
Quercetin (**8**)	/301	301, 273, 179, 153	[24, 25]
Kaempferol (**9**)	/287	287, 258, 165, 153, 121	[26, 27]

**Table 2:** Regression data, limit of detection (LOD), and limit of quantification (LOQ) for the five external standards.

Standard	Calibration curve	*r* ^2^	LOD	LOQ	Recovery (%) ± RSD	
			(mg/L)	(mg/L)	50 mg/L	100 mg/L
Gallic acid	*y* = 25.171*x* + 335.04	0.9993	0.032	0.107	79.6 ± 6.1	85.2 ± 0.5
Protocatechuic acid	*y* = 37.614*x* + 1137.3	0.9982	0.030	0.102	88.5 ± 0.2	87.8 ± 0.5
7-Hydroxycoumarin	*y* = 10.873*x* − 28.39	0.9955	0.142	0.473	102.8 ± 0.7	98.6 ± 11.6
Quercetin 3-*O*-glucoside	*y* = 30.166*x* + 267.56	0.9949	0.037	0.125	76.1 ± 0.1	90.9 ± 14.0
Kaempferol	*y* = 39.493*x* + 1475.4	0.9907	0.028	0.094	100.0 ± 3.0	97.4 ± 1.4

*y*: peak area of standard; *x*: concentration of standard (mg/L).

**Table 3:** Concentration of polyphenols in *Euphorbia supina* (mg/kg fresh plant).

Compounds	Mean ± SD
Gallic acid (**1**)	264.4 ± 0.7
Protocatechuic acid (**2**)	124.3 ± 0.3
Nodakenin (**3**)	120.0 ± 4.7
Quercetin 3-*O*-hexoside (**4**)	458.3 ± 4.9
Quercetin 3-*O*-pentoside (**5**)	1648.2 ± 20.2
Kaempferol 3-*O*-hexoside (**6**)	553.8 ± 4.2
Kaempferol 3-*O*-pentoside (**7**)	126.9 ± 1.5
Quercetin (**8**)	118.7 ± 1.0
Kaempferol (**9**)	21.0 ± 4.6

Total	3352.9 ± 2.8

**Table 4:** Antioxidant activity (%).

	Concentration, mg/L					Scavenging activity
	25	50	100	200	500	
DPPH	29.85 ± 0.86^a^	31.75 ± 1.41^a^	40.88 ± 1.15^b^	47.63 ± 1.93^c^	71.44 ± 1.04^d^	229.19 ± 22.34^A^
ABTS	12.25 ± 0.67^a^	19.76 ± 0.63^b^	32.38 ± 0.46^c^	53.83 ± 0.27^d^	88.13 ± 0.73^e^	180.94 ± 3.48^A^
RP	0.091 ± 0.001^a^	0.108 ± 0.002^b^	0.132 ± 0.002^c^	0.178 ± 0.001^d^	0.328 ± 0.001^e^	443.60 ± 4.01^B^

Assay wavelength: 1,1-diphenyl-2-picrylhydrazyl (DPPH) = 517 nm and 2,2′-azinobis(3-ethylbenzothiazoline-6-sulfonic acid) (ABTS) = 414 nm, and reducing power (RP) = 700 nm.

Butylated hydroxytoluene (BHT) EC_50_; DPPH: 121.85 ± 0.39 mg/L; ABTS: 93.85 ± 0.43 mg/L; RP: 26.71 ± 0.69 mg/L.

Each value represents mean ± standard deviation (SD), *n* = 5.

^a–e^Means with different superscripts in the row are significantly different at *P* < 0.05.

^A^EC_50 _(mg/L) values were calculated from the calibration curves using five different concentrations (25–500 mg/L) in quintuplicate and their data were presented as 50% scavenging activity.

^B^RP value (EC_0.3_) was reducing activity calculated from the calibration curves using five different concentrations (25–500 mg/L) in quintuplicate.

## References

[1] Prostrate Spurge Description http://www.turf.uiuc.edu/weed_web/descriptions/prostratespurge.htm.

[2] Tanaka R, Kurimoto M, Yoneda M, Matsunaga S (1990). 17*β*,21*β*-Epoxyhopan-3*β*-ol and *β*-alnincanol from *Euphorbia supina*. *Phytochemistry*.

[3] An R-B, Kwon J-W, Kwon T-O, Chung W-T, Lee H-S, Kim Y-C (2007). Chemical constituents from the whole plants of *Euphorbia supina* Rafin. *Korean Journal of Pharmacognosy*.

[4] Tanaka R, Matsunaga S (1989). Loliolide and olean-12-en-3*β*,9*α*,11*α*-triol from *Euphorbia supina*. *Phytochemistry*.

[5] Agata I, Hatano T, Nakaya Y (1991). Tannins and related polyphenols of euphorbiaceous plants. VIII. Eumaculin A and eusupinin A, and accompanying polyphenols from *Euphorbia maculata* L. and *E. supina* Rafin. *Chemical and Pharmaceutical Bulletin*.

[6] Lee S-H, Tanaka T, Nonaka G, Nishioka I (1991). Tannins and related compounds. CV. Monomeric and dimeric hydrolyzable tannins having a dehydrohexahydroxydiphenoyl group, supinanin, euphorscopin, euphorhelin and jolkianin, from *Euphorbia* species. *Chemical and Pharmaceutical Bulletin*.

[7] Fang Z, Zeng X, Zhang Y, Zhou G (1993). Chemical constituents of spotted leaf euphorbia (*Euphorbia supina*). *Zhongcaoyao*.

[8] Erlund I (2004). Review of the flavonoids quercetin, hesperetin, and naringenin. Dietary sources, bioactivities, bioavailability, and epidemiology. *Nutrition Research*.

[9] Le Marchand L (2002). Cancer preventive effects of flavonoids—a review. *Biomedicine and Pharmacotherapy*.

[10] Xu YC, Leung SWS, Yeung DKY (2007). Structure-activity relationships of flavonoids for vascular relaxation in porcine coronary artery. *Phytochemistry*.

[11] Treutter D (2006). Significance of flavonoids in plant resistance: a review. *Environmental Chemistry Letters*.

[12] Nierheilig H, Piché Y (2002). Signalling in arbuscular mycorrhiza: facts and hypotheses. *Flavonoids in Cell Function*.

[13] Hong HK, Kwak JH, Kang SC (2008). Antioxidative constituents from the whole plants of *Euphorbia supina*. *Korean Journal of Pharmacognosy*.

[14] de Rijke E, Out P, Niessen WMA, Ariese F, Gooijer C, Brinkman UAT (2006). Analytical separation and detection methods for flavonoids. *Journal of Chromatography A*.

[15] Kim HG, Kim G-S, Park S (2012). Flavonoid profiling in three citrus varieties native to the Republic of Korea using liquid chromatography coupled with tandem mass spectrometry: contribution to overall antioxidant activity. *Biomedical Chromatography*.

[16] Seo ON, Kim G-S, Park S (2012). Determination of polyphenol components of *Lonicera japonica* Thunb. Using liquid chromatography-tandem mass spectrometry: contribution to the overall antioxidant activity. *Food Chemistry*.

[17] Chen L, Qi J, Chang Y-X, Zhu D, Yu B (2009). Identification and determination of the major constituents in Traditional Chinese Medicinal formula Danggui-Shaoyao-San by HPLC-DAD-ESI-MS/MS. *Journal of Pharmaceutical and Biomedical Analysis*.

[18] Liu X, Jiang S, Xu K (2009). Quantitative analysis of chemical constituents in different commercial parts of Notopterygium incisum by HPLC-DAD-MS. *Journal of Ethnopharmacology*.

[19] Ek S, Kartimo H, Mattila S, Tolonen A (2006). Characterization of phenolic compounds from lingonberry (*Vaccinium vitis-idaea*). *Journal of Agricultural and Food Chemistry*.

[20] Lhuillier A, Fabre N, Moyano F (2007). Comparison of flavonoid profiles of *Agauria salicifolia *(Ericaceae) by liquid chromatography-UV diode array detection-electrospray ionisation mass spectrometry. *Journal of Chromatography A*.

[21] Kumar N, Bhandari P, Singh B, Bari SS (2009). Antioxidant activity and ultra-performance LC-electrospray ionization-quadrupole time-of-flight mass spectrometry for phenolics-based fingerprinting of Rose species: *Rosa damascena*, *Rosa bourboniana* and *Rosa brunonii*. *Food and Chemical Toxicology*.

[22] Olszewska M (2007). Quantitative HPLC analysis of flavonoids and chlorogenic acid in the leaves and inflorescences of *Prunus serotina* Ehrh. *Acta Chromatographica*.

[23] Hong HK, Kwak JH, Kang SC (2008). Antioxidative constituents from the whole plants of *Euphorbia supina*. *Korean Journal of Pharmacognosy*.

[24] Hong Y-J, Mitchell AE (2004). Metabolic profiling of flavonol metabolites in human urine by liquid chromatography and tandem mass spectrometry. *Journal of Agricultural and Food Chemistry*.

[25] Meng X, Maliakal P, Lu H, Lee M-J, Yang CS (2004). Urinary and plasma levels of resveratrol and quercetin in humans, mice, and rats after ingestion of pure compounds and grape juice. *Journal of Agricultural and Food Chemistry*.

[26] Dehkharghanian M, Adenier H, Vijayalakshmi MA (2010). Study of flavonoids in aqueous spinach extract using positive electrospray ionisation tandem quadrupole mass spectrometry. *Food Chemistry*.

[27] Murota K, Terao J (2003). Antioxidative flavonoid quercetin: implication of its intestinal absorption and metabolism. *Archives of Biochemistry and Biophysics*.

[28] Calderón-Montaño JM, Burgos-Morón E, Pérez-Guerrero C, López-Lázaro M (2011). A review on the dietary flavonoid kaempferol. *Mini-Reviews in Medicinal Chemistry*.

[29] Oxidative stress http://en.wikipedia.org/wiki/Oxidative_stress.

[30] Hopps E, Noto D, Caimi G, Averna MR (2010). A novel component of the metabolic syndrome: the oxidative stress. *Nutrition, Metabolism and Cardiovascular Diseases*.

[31] Finkel T (2005). Radical medicine: treating ageing to cure disease. *Nature Reviews Molecular Cell Biology*.

[32] Valko M, Leibfritz D, Moncol J, Cronin MTD, Mazur M, Telser J (2007). Free radicals and antioxidants in normal physiological functions and human disease. *International Journal of Biochemistry and Cell Biology*.

[33] Lee JH, Lee SJ, Park S (2011). Characterisation of flavonoids in *Orostachys japonicus* A. Berger using HPLC-MS/MS: contribution to the overall antioxidant effect. *Food Chemistry*.

[34] Mosmann T (1983). Rapid colorimetric assay for cellular growth and survival: application to proliferation and cytotoxicity assays. *Journal of Immunological Methods*.

